# Editorial: Molecular basis of asexual reproduction and its application in crops

**DOI:** 10.3389/fpls.2022.1033212

**Published:** 2022-10-04

**Authors:** Huihui Guo, Xiushan Qi, Tongtong Li, Yijie Fan, Heqiang Huo, Qingyi Yu, Fanchang Zeng

**Affiliations:** ^1^ State Key Laboratory of Crop Biology, College of Agronomy, Shandong Agricultural University, Tai’an, China; ^2^ Department of Environmental Horticulture, University of Florida, Apopka, FL, United States; ^3^ Texas A&M AgriLife Research, Texas A&M University, Dallas, TX, United States

**Keywords:** asexual reproduction, plant regeneration, regulation and application, apomixis, genes identification

Plants display a remarkable capacity for somatic cell plasticity for asexual reproduction. This notable example of totipotency is demonstrated by plant cells that can develop into embryos and then complete plants (organogenesis, embryogenic differentiation, somatic embryogenesis, plant regeneration *in vitro*, and apomixis *in vivo*). Asexual reproduction is an additional unique reproduction mode of plants relative to traditional sexual reproduction. The innovation of breeding and propagation technology is the key to and an imperative segment of the competition in the crop breeding industry. The asexual reproduction approach is an important direction in the development of modern cell engineering and biotechnology breeding and has great development potential for crop artificial seed and plant factories. However, at present, the regulation principle and molecular mechanism of asexual reproduction and propagation are not clear, and the related knowledge and biotechnology are insufficient. In this Research Topic, we aimed to gather new research, hypotheses, and reviews that would help to better understand the progress and prospects of crop asexual reproduction to promote theoretical and technological innovation in this field. In this way, a series of articles provides an overview of the discoveries in the field of asexual reproduction and its potential applications in crops.

This Research Topic features a number of Original Research Articles. Somatic embryogenesis and plant regeneration are the developmental reprogramming of somatic cells toward embryogenesis, and they form the cornerstone of asexual reproduction. A series of cellular processes and molecular events occur during plant embryogenesis, such as somatic dedifferentiation, cell division initiation, gene expression pattern reprogramming, and metabolism changes. Revealing the underlying cellular and molecular basis would strengthen our understanding of plant embryonic differentiation initiation during somatic embryogenesis and plant regeneration for asexual reproduction. Fan et al. report that dynamic transcriptome analysis reveals a complex regulatory pathway underlying the induction and dose effect of different exogenous auxins IAA and 2,4-D during *in vitro* crop embryogenic redifferentiation. They demonstrate a systematic molecular response to auxin signals and the molecular pathway that regulates embryogenic redifferentiation during somatic embryogenesis. Based on the results, the broad repertoire of genes and complex expression patterns in somatic embryogenesis suggest the combinatorial multiple cellular pathways controlled by a concerted gene regulatory network underlie somatic embryogenesis.

Embryogenesis and reproduction are not strictly dependent on fertilization in plants, which can produce embryos in seeds asexually, a process known as apomixis. Investigations on the molecular basis and identification of functional genes involved in apomixis *in vivo* are of great fundamental and practical importance in the highly efficient generation of clonal seeds by asexual reproduction. The study by Chahal et al. investigates the parthenogenetic potential of three phylogenetically distant BABY BOOM (BBM) transgenes from *S. italica*, each a member of a different phylogenetic BBM clade. All *SiBBM* transgenes induced various levels of parthenogenetic embryo development, resulting in viable haploid T1 seedlings in rice.

The development of a visual labeling system is a powerful and useful tool for *in vitro* cell manipulation as well as genetic transformation when used in combination with modern molecular biotechnology in plants. Hu et al. report an optimized protein fluorescence reporting system for somatic embryogenesis regeneration screening and visual labeling of functional genes in the cotton genetic transformation process. It provides a visual marker for labeling transgenic somatic embryos using the fluorescence reporting system based on the screening of 11 fluorescent proteins. The optimized fluorescence labeling system in this study offers the potential for accelerating somatic cell regeneration efficiency and the *in vivo* monitoring of diverse cellular processes. In addition, the study by Nie et al. successfully induced calli from two diploid cotton species, *Gossypium. raimondii* and *Gossypium. sturtianum*, and transcriptome sequencing analysis was performed on the calli to reveal new genes responding to salt stress, which will lay the foundation for plant regeneration and protoplast fusion.

Dynamic balance (e.g., auxin and cytokinin make for proembryogenic masses (PEMs), ethylene for preglobular embryos, GA for globular embryos, and ABA for mature embryos) and interactions with hormones play a pivotal role in switching cell fate during somatic embryogenesis developmental plasticity of asexual reproduction. They can be considered vital regulators of developmental switching during the totipotency of somatic plant cells. While emphasizing the endogenous signal of the switch, exogenous plant growth regulators are also fundamental. Chen et al. report that uniconazole augments abscisic acid in promoting somatic embryogenesis in *Gossypium hirsutum*. The study suggests that uniconazole could modulate callus proliferation and callus differentiation rate by regulating the endogenous levels of IAA and ABA.

Moreover, the Leafy cotyledon (LEC), Wuschel (WUS), Baby boom (BBM), Monooxygenase, and somatic embryogenesis receptor kinase (SERK) identified may serve as markers to distinguish embryogenic cells, enabling an early diagnosis of embryogenic potential. Wang et al. demonstrate that GhLBDs (LATERAL ORGAN BOUNDARIES DOMAIN transcription factors in *Gossypium hirsutum*) promote callus initiation and act as selectable markers to increase transformation efficiency. This study provides new insights into callus initiation regulatory mechanisms and strategies for improving transformation efficiency in crops.

The Research Topic also features Review Articles focusing on asexual reproduction. Joshi et al. review the promotion role and molecular mechanism of AGAMOUS-Like15 (AGL15) in somatic embryogenesis, focusing on the MADS-domain transcription factor AGL15, and show a positive correlation between accumulation levels and the capacity for somatic embryogenesis. The review relates AGL15 function to other transcription factors, hormones, and epigenetic modifiers involved in somatic embryo development. Yin et al. review the options for engineering apomixis in plants. This review summarizes the current understanding of apomixis and highlights the successful introduction of apomixis methods into sexual crops. They also summarize three schemes to achieve engineered apomixis, which will offer more opportunities for the realization of apomictic reproduction.

Taken together, these articles provide an overview of the molecular basis and regulation of asexual reproduction, including embryonic differentiation *in vitro*, somatic embryogenesis, and apomixis *in vivo*. As the basis for plant cell engineering and biotechnology applications, asexual reproduction is of great practical importance in plant factories and modern breeding. It is imperative to unravel the regulatory mechanisms of asexual reproduction and developmental flexibility for potential applications in crops, but a long and difficult road is ahead.

## Author contributions

All authors listed have made a substantial and intellectual contribution to the work and approved it for publication.

## Funding

National Key Research and Development Program (2021YFF1001203), the Agricultural Seed Project of Shandong Province (2020LZGC002), and the National Natural Science Foundation of China (32001589).

## Acknowledgments

We would like to thank all authors for their contributions to this Research Topic. We are also grateful to all reviewers for their evaluation of the manuscripts submitted.

## Conflict of interest

The authors declare that the research was conducted in the absence of commercial or financial relationships as a potential conflict of interest.

## Publisher’s note

All claims expressed in this article are solely those of the authors and do not necessarily represent those of their affiliated organizations, or those of the publisher, the editors and the reviewers. Any product that may be evaluated in this article, or claim that may be made by its manufacturer, is not guaranteed or endorsed by the publisher.

